# Supplementary material for: Use of volatile agents for sedation in the intensive care unit: A national survey in France
Source: PLoS One. 2021 Apr 15;16(4):e0249889. doi: 10.1371/journal.pone.0249889 (PMC8049230; doi:10.1371/journal.pone.0249889)
Supplement: S1 File — (DOCX) [file pone.0249889.s001.docx]

**Use of volatile agents for sedation in the intensive care unit: A national survey in France**

Raiko Blondonnet, Audrey Quinson, Céline Lambert, Jules Audard, Thomas Godet, Ruoyang Zhai, Bruno Pereira, Emmanuel Futier, Jean-Etienne Bazin, Jean-Michel Constantin, Matthieu Jabaudon

##

## **Additional files**

**Additional file: Supplemental Content 1.** Survey questionnaire

1. **General characteristics of the ICU**

Your place of professional exercise:

- University Hospital
- General Hospital
- Private health structure
- Other

In which type of intensive care unit do you work?

- Medico-surgical / Polyvalent
- Medical
- Step-down unit
- Medical intensive care, such as a coronary care unit
- Other

In which French region do you work?

- Auvergne - Rhône-Alpes
- Bourgogne-Franche-Comté
- Bretagne
- Centre-Val de Loire
- Corse
- Grand-Est
- Hauts-de-France
- Ile-de-France
- Normandie
- Nouvelle-Aquitaine
- Occitanie
- Pays de la Loire
- Provence-Alpes-Côte d’Azur
- Corse
- DOM-TOM

Number of senior physicians in your unit (full-time equivalent): (numerical value)

Number of residents in your unit: (numerical value)

Number of beds in your intensive care unit: (numerical value)

Number of step-down beds in your unit: (numerical value)

Number of patients admitted per year in your intensive care unit: (numerical value)

What percentage of patients are admitted with ARDS in your unit? (numerical percentage value)

What is the average length of stay in the intensive care unit (in days) of your patients? (numerical value)

What is the average duration (in days) of invasive mechanical ventilation in your patients? (numerical value)

What is the mortality rate, in the intensive care unit, of your patients? (numerical percentage value)

1. **General data on inhaled sedation use**

Are you familiar with inhaled ICU sedation?

- No
- Yes:
  - I am familiar with the AnaConDa System
  - I am familiar with the Mirus System
  - I am familiar with another system

Do you have dedicated systems to deliver inhaled sedation available in your institution?

- No

If not, have you ever borrowed a ventilator from the operating room to deliver halogenated agents to your ICU patients?

🗆 Yes

🗆 No

- Yes:
  - The Anaconda system
  - The Mirus system
  - Another system

If yes, how long have you been using this(these) system(s)?

- less than a year
- 1-5 years
- 5-10 years
- >10 years

Do you use inhaled sedation in your ICU?

- Never
- Sometimes
- Often

Who uses inhaled sedation in your ICU?

- Nobody
- Some physicians
- All physicians

In how many patients per year do you use inhaled ICU sedation?

- <20
- 20-50
- 50-100
- >100

If you do not use inhaled ICU sedation, what are the reasons? (multiple answers possible)

- No equipment available
- Untrained medical staff
- Untrained paramedical personnel
- No obvious interest
- Heavy organization
- Concerns about air pollution risk or ecological impact
- Undesirable effects
- Lack of habit
- Cost issues

If you use inhaled sedation in your ICU, what is/are the indication(s) for use? (multiple answers possible

- Medical
- Post-surgery
- ARDS
- Traumatology
- Failure of IV sedation (drug or alcohol addicts, etc.)
- Asthma
- Cardioprotection
- Status epilepticus
- Neuroprotection
- No specific indication
- Other(s)

In your opinion, what are the potential benefits of inhaled ICU sedation? (multiple answers possible)

- I don't know
- Ease of use, including “on/off” effects
- Bronchodilating properties
- Anti-inflammatory properties
- Low cost
- Other(s)

1. **Practical aspects of inhaled ICU sedation**

Do you have a written protocol for inhaled sedation in your ICU?

- No
- Yes

Do you have any specific training in inhaled ICU sedation?

- No
- Yes

What halogenated agent do you use? (multiple answers possible)

- Sevoflurane
- Isoflurane
- Desflurane

For what reason(s) do you use this (these) halogenated agent(s)? (multiple answers possible)

- Availability
- Cost
- Ease of use
- Low metabolism
- Other(s)

With which mode(s) of ventilation do you use inhaled sedation? (multiple answers possible)

- Volume-controlled or pressure-controlled ventilation
- Pressure support (invasive) ventilation
- Pressure support (noninvasive) ventilation
- Other(s)

Do you usually combine an opioid agent with inhaled sedation?

- No
- Yes

If yes, which one(s)? (multiple answers possible)

- Sufentanil
- Fentanyl
- Remifentanil
- Other(s)

Do you usually combine another sedative agent when you use inhaled ICU sedation?

- No
- Yes

If yes, which one(s)? (multiple answers possible)

- Midazolam
- Propofol
- Ketamine
- Dexmedetomidine
- Other(s)

With what exhaled fraction of halogenated agents do you usually initiate inhaled ICU sedation?

- For sevoflurane: (numerical value)
- For isoflurane: (numerical value)
- For desflurane: (numerical value)

What expired fraction of halogenated agents do you typically target for "deep" ICI sedation?

- For sevoflurane: (numerical value)
- For isoflurane: (numerical value)
- For desflurane: (numerical value)
- Non applicable
- No expired fraction is targeted, I adapt sedation based on a score
- I only set a maximal expired fraction for safety reasons

How do you usually monitor the depth of sedation? (multiple answers possible)

- Sedation scale such as the RASS
- Monitoring of expired gas fraction
- Use of the Bispectral index (BIS)
- Other(s) :

Do you usually perform plasma assays for halogenated agents and/or their derivatives?

- No
- Yes

When do you usually stop inhaled sedation?

- When weaning off the ventilator
- During extubation
- Systematically at 48h
- No specific time limit
- Other(s)

In your opinion, what are the absolute contraindications of inhaled sedation?

- I don't know
- Acute renal failure
- Liver failure
- Pregnancy
- History of malignant hyperthermia
- Intracranial hypertension
- Other(s)

Have you ever had any serious adverse events that you believe were caused by inhaled sedation in ICU patients?

- No
- Yes

If yes, please detail: (multiple answers possible)

- Malignant hyperthermia
- Acute renal failure
- Severe respiratory acidosis
- Other(s)

What is your overall satisfaction with inhaled ICU sedation?

- Not satisfied at all
- Unsatisfied
- Indifferent
- Satisfied
- Very satisfied

In your opinion, is inhaled sedation an interesting alternative to intravenous sedation in the intensive care setting?

- No
- Yes

Free comments, if any:

**Additional file: Supplemental Content 2.** General characteristics of survey respondents (N=187). D*ata are presented as numbers (with associated percentages) or as medians [interquartile ranges]. ARDS: Acute Respiratory Distress Syndrome, ICU: Intensive Care Unit.*

| N=187 | |
| --- | --- |
| Type of hospital, n (%) | |
| Teaching hospital | 45 (24) |
| General hospital | 111 (59) |
| Private medical center | 27 (14) |
| Military hospital | 4 (2) |
| Type of ICU, n (%) | |
| Polyvalent | 170 (91) |
| Medical | 15 (8) |
| Burn center | 2 (1) |
| Number of physicians per ICU, n | |
| Senior physicians | 6 [5-7.3] |
| Residents | 4 [2-6] |
| Number of beds per ICU, | |
| ICU beds | 12 [8.5-15] |
| Step-down units beds | 6 [4-8] |
| ICU beds/senior | 1.9 [1.6-2.5] |
| Patients per year, n | 600 [417-850] |
| Patients with ARDS (%) | 9 [4-15] |
| Duration of ICU stay (days) | 6.7 [5.8-8] |
| Duration of mechanical ventilation (days) | 6 [4-8] |
| Mortality (%) | 20 [18-24]¶ |

**Additional file: Supplemental Content 3.** Geographical distribution and epidemiological data on respondents (n=187). *DOM-TOM: Département d’outre-mer-Territoire d’outre-mer.*

| **French region** | **Number of ICU** | **%** |
| --- | --- | --- |
| Auvergne Rhône-Alpes | 25 | 13.4 |
| Bourgogne Franche-Comté | 8 | 4.3 |
| Bretagne | 7 | 3.7 |
| Centre Val de Loire | 5 | 2.7 |
| Corse | 2 | 1.1 |
| DOM-TOM | 1 | 0.5 |
| Grand Est | 18 | 9.6 |
| Hauts-de-France | 16 | 8.6 |
| Ile-de-France | 40 | 21.4 |
| Normandie | 5 | 2.7 |
| Nouvelle Aquitaine | 11 | 5.9 |
| Occitanie | 13 | 7.0 |
| Pays de la Loire | 7 | 3.7 |
| Provence Alpes Côte-d'Azur | 29 | 15.5 |

**Additional file: Supplemental Content 4.** Geographical distribution and epidemiological data of non-respondents (n=187, excluding pediatric intensive care units). *DOM-TOM: Département d’outre-mer-Territoire d’outre-mer.*

| **French region** | **Number of ICU** | **%** |
| --- | --- | --- |
| Auvergne Rhône-Alpes | 18 | 9,6 |
| Bourgogne Franche-Comté | 7 | 3,7 |
| Bretagne | 3 | 1,6 |
| Centre Val de Loire | 7 | 3,7 |
| Corse | 0 | 0,0 |
| DOM-TOM | 9 | 4,8 |
| Grand Est | 20 | 10,7 |
| Hauts-de-France | 17 | 9,1 |
| Ile-de-France | 44 | 23,5 |
| Normandie | 13 | 7,0 |
| Nouvelle Aquitaine | 13 | 7,0 |
| Occitanie | 21 | 11,2 |
| Pays de la Loire | 2 | 1,1 |
| Provence Alpes Côte-d'Azur | 13 | 7,0 |
